# Maternal occupational exposure and congenital heart defects in offspring

**DOI:** 10.5271/sjweh.3912

**Published:** 2020-10-30

**Authors:** Nynke Spinder, Jorieke EH Bergman, Hans Kromhout, Roel Vermeulen, Nicole Corsten-Janssen, H Marike Boezen, Gideon J du Marchie Sarvaas, Hermien EK de Walle

**Affiliations:** 1 University of Groningen, University Medical Center Groningen, Department of Epidemiology, Groningen, The Netherlands; 2 University of Groningen, University Medical Center Groningen, Department of Genetics, Groningen, The Netherlands; 3 Institute for Risk Assessment Science, Division of Environmental Epidemiology, Utrecht University, The Netherlands; 4 University of Groningen, University Medical Center Groningen, Groningen Research Institute for Asthma and COPD (GRIAC), Groningen, The Netherlands; 5 University of Groningen, University Medical Center Groningen, Department of Paediatric Cardiology, Groningen, The Netherlands

**Keywords:** maternal characteristic, maternal exposure, metal gas, metal fume, mineral dust, mother, organic dust, pesticide, solvent, work

## Abstract

**Objectives:**

Congenital heart defects (CHD) are the most prevalent congenital anomalies. This study aims to examine the association between maternal occupational exposures to organic and mineral dust, solvents, pesticides, and metal dust and fumes and CHD in the offspring, assessing several subgroups of CHD.

**Methods:**

For this case–control study, we examined 1174 cases with CHD from EUROCAT Northern Netherlands and 5602 controls without congenital anomalies from the Lifelines cohort study. Information on maternal jobs held early in pregnancy was collected via self-administered questionnaires, and job titles were linked to occupational exposures using a job exposure matrix.

**Results:**

An association was found between organic dust exposure and coarctation of aorta [adjusted odds ratio (OR_adj_) 1.90, 95% confidence interval (CI) 1.01–3.59] and pulmonary (valve) stenosis in combination with ventricular septal defect (OR_adj_ 2.68, 95% CI 1.07–6.73). Mineral dust exposure was associated with increased risk of coarctation of aorta (OR_adj_ 2.94, 95% CI 1.21–7.13) and pulmonary valve stenosis (OR_adj_ 1.99, 95% CI 1.10–3.62). Exposure to metal dust and fumes was infrequent but was associated with CHD in general (OR_adj_ 2.40, 95% CI 1.09–5.30). Exposure to both mineral dust and metal dust and fumes was associated with septal defects (OR_adj_ 3.23, 95% CI 1.14–9.11). Any maternal occupational exposure was associated with a lower risk of aortic stenosis (OR_adj_ 0.32, 95% CI 0.11–0.94).

**Conclusions:**

Women should take preventive measures or avoid exposure to mineral and organic dust as well as metal dust and fumes early in pregnancy as this could possibly affect foetal heart development.

Congenital heart defects (CHD) are the most prevalent congenital anomalies. Approximately 7 per 1000 pregnancies are affected by a CHD (1). Of these, >90% are live births, ~8% of the pregnancies are terminated because of CHD, and 1–2% are still births (1). Since the introduction of prenatal ultrasound screening, ~50% of critical CHD cases are detected prenatally, and this number continues to increase with improvements in ultrasound technology, recommendations, and training for foetal heart examination (2). Survival rates are also increasing due to improved surgical intervention and intensive care (3). Major CHD have a significant impact on children’s physical and mental health in the short- and long-term (4, 5), making it important to identify modifiable risk factors to prevent CHD in offspring.

Both genetic and environmental factors are involved in the development of CHD. Chromosomal anomalies are found in 12% of the infants with CHD (6), and an increasing number of gene point mutations have been identified that cause isolated non-syndromic CHD (7). Having first-degree family members with CHD or a multiple pregnancy increases the risk of CHD in offspring by 1–10% (8). In addition, certain maternal illnesses (eg, maternal diabetes, phenylketonuria, rubella infection), exposure to specific medications during pregnancy (eg, anticonvulsants and higher doses of lithium), and high maternal weight increase the risk of CHD in offspring (8, 9). Lifestyle factors such as parental smoking and alcohol use can also increase the risk of CHD (8–10), while periconceptional folic acid supplementation decreases this risk (11). Other risk factors include exposure to environmental agents such as ambient air pollution, chemicals, and metals (12, 13).

Exposure to potential teratogenic agents can occur in the workplace. A recent meta-analysis found an association between maternal occupational exposure to solvents and CHD (14). In this meta-analysis, it was not possible to examine subgroups of CHD since the majority of studies selected included small numbers of cases. However, it is important to assess subgroups of CHD as defects differ in etiology and develop during different stages of embryogenesis. The aim of the present study was to examine the association between various types of maternal occupational exposures early in pregnancy and subgroups of CHD in the offspring.

## Methods

### Study design

Cases were selected from the European Registration of Congenital Anomalies and Twins Northern Netherlands (EUROCAT NNL). This registry collects data of infants born with a congenital anomaly in the three northern provinces of The Netherlands. In addition to live-born infants (up to 10 years of age at notification), EUROCAT NNL registers stillbirths, miscarriages, and terminated pregnancies affected by a congenital anomaly. EUROCAT NNL identifies eligible cases by active case ascertainment using hospital records, prenatal diagnosis records, and postmortem records. After parents give informed consent, they are asked to complete a questionnaire. Information is collected regarding the pregnancy, obstetric and medical history, demographic characteristics, use of medication, and occupation and lifestyle factors early in pregnancy (15).

Controls without congenital anomalies (non- malformed controls) were selected from the Lifelines cohort. Lifelines is a three-generation cohort study following 167 000 participants over a 30-year period in the same geographical region as EUROCAT NNL. Lifelines participants were recruited through their general practitioners, and participants (18–65 years old) were also asked to invite their offspring and parents in order to create a three-generation cohort. Participants’ children could participate if they were between 6 months and 18 years old. Parents of participating children completed a questionnaire regarding the pregnancy, their health during pregnancy, childbirth, and the child’s health in the first six months of life (16).

### Case and control definition

**Figure 1:**
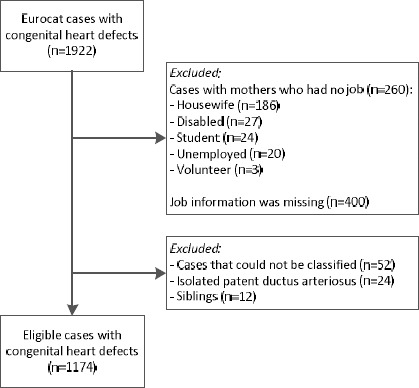
Flow chart case selection from EUROCAT North Netherlands.

CHD cases were coded by trained registry staff according to the International Classification of Diseases 9^th^ revision (ICD-9) until 2001 and according to ICD 10^th^ revision (ICD-10) from 2002 onwards, using international EUROCAT guidelines (17, 18). Cases with heterotaxy syndrome or an underlying genetic, chromosomal, or syndromic condition were excluded, resulting in the selection of 1922 CHD cases born 1997– 2013 (figure 1). Mothers with missing job information (N=400) or without a job (N=260) were excluded to avoid healthy worker bias.

Three of the study authors classified the remaining cases according to the Botto classification to account for the diversity of cardiac phenotypes and underlying developmental mechanisms. The Botto classification has been described previously (19). Briefly, morphologically homogeneous groups were produced for each cardiac phenotype, based on anatomy and developmental and epidemiologic evidence. The seven main heart defect groups were: conotruncal heart defects, atrioventricular septal defects (AVSD), anomalous pulmonary venous return (APVR), left ventricular outflow tract obstruction (LVOTO), right ventricular outflow tract obstruction (RVOTO), septal defects, and complex heart defects. A few cardiac malformations are not included in the Botto classification. In line with the classification described by Riehle-Collarusso and colleagues (20), cases with a bicuspid aorta valve were classified as LVOTO anomaly and cases with a vascular ring (vascular rings/slings, double aortic arch, right descending aortic arch, aberrant left subclavian artery, or pulmonary artery sling) were classified as conotruncal defects. Cases were excluded if they could not be classified (eg, coronary artery malformations, N=52) or constituted isolated patent ductus arteriosus (N=24). Additionally, CHD were classified as isolated defect (only the heart is affected) or as multiple defect (presence of cardiac and extra-cardiac malformations). Cases were also classified by the complexity of their cardiac phenotype: simple (anatomically discrete or well-recognized single entities), association (common, uncomplicated combinations of heart defects), and complex malformations (those not described as simple or association). If multiple siblings were affected by a CHD, one infant per family was randomly selected to avoid genetic correlation, resulting in exclusion of 12 cases. Overall, 1174 infants with CHD were included; 85.4% of these infants were live-born, 10.6% were live-born but died after birth, 2.6% were terminated pregnancies, 1.1% were stillborn infants, and 0.3% were miscarriages.

**Figure 2:**
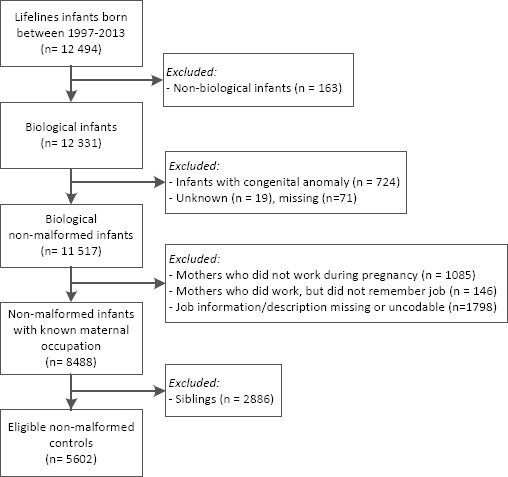
Flow chart control selection from Lifelines.

All participants from the Lifelines cohort born 1997–2013 (same years as the EUROCAT NNL cases) were selected as controls (N=12 494, figure 2). Only infants of which the biological mother was a Lifelines participant were included (N=12 331). We excluded 814 infants because one or more congenital anomalies were reported or information on congenital anomalies was missing. As with cases, mothers without a job or missing job information were excluded (N=3029) and only one infant per family was selected, resulting in exclusion of another 2886 infants. In total, 5602 children without congenital anomalies were included as the control group.

### Exposure assessment

Two authors coded the mother’s description of her job early in pregnancy using the International Standard Classification of Occupations 1988 (ISCO88) (21), without knowledge of case or study details. To translate ISCO88 codes into occupational exposure, the ALOHA+ job exposure matrix (JEM) was used. Occupational exposure was assigned based on six categories: organic and mineral dust, solvents, pesticides, metal dust and fumes, and gases and fumes. Those categories were combined into one category which is referred to as “any” exposure. All women exposed to one or more exposure categories were labelled as exposed to any exposure. This JEM assigns exposure intensity in three categories (no, low, and high exposure). Because “high” (intensity and probability) exposure did not occur often, the categories “low” and “high” were combined into “exposed”. The ALOHA+ JEM is specifically built for use in general population studies (22, 23). However, in our female study population, there was a strong correlation of exposure to solvents with exposure to gases and fumes and to organic dust with gases and fumes (Spearman’s rank correlation coefficient = 0.75 and 0.80, respectively). Therefore, the association of gases and fumes with CHD was not analyzed.

### Statistical analysis

Baseline characteristics of mothers and infants were tabulated, and differences between cases and controls were tested for significance using Chi-square tests. The following covariates were assessed: child sex (male/female), birth year (1997–2000, 2001–2004, 2005–2008, or 2009–2013), maternal age at delivery (15–19, 20–24, 25–29, 30–34, 35–39, or ≥40 years old), maternal body mass index (BMI) (self-reported pre-pregnancy weight and height for EUROCAT NNL cases and objective measurement at baseline visit for Lifelines controls) [underweight (<18.5 kg/m^2^), normal (18.5–24.9 kg/m^2^), overweight (25.0–29.9 kg/m^2^), or obese (≥30 kg/m^2^)], maternal education level [low (primary school, lower vocational education, pre-vocational education), middle (secondary vocational education, general secondary education or pre-university education), or high (higher professional education or academic education)], maternal smoking and alcohol use, folic acid use (no/not during periconceptional period, yes/sometime during periconceptional period), and fertility problems [no, yes (self-reported fertility problems and/or fertility treatment)].

The association between maternal occupational exposure early in pregnancy and CHD was assessed using univariate and multivariate logistic regression analysis to estimate crude and adjusted odd ratios (OR_crude_/OR_adj_). The multivariate logistic regression associations were adjusted for child sex, maternal age at delivery, maternal educational level, maternal BMI, smoking and alcohol use during pregnancy, folic acid supplementation, and fertility problems, based on Chi-square tests (table 1). Although the correlation between exposure to mineral dust and exposure to metal dust and fumes was negligible (Spearman’s rank correlation coefficient = 0.08), exposure to metal dust and fumes contributes to mineral dust exposure. Consequently, additional analyses were performed with a combination of those exposures. Stratified analyses were performed for cases with isolated and multiple defects. In addition, a sensitivity analysis restricted to non-smoking mothers who did not report drinking alcohol early in pregnancy was performed to explore the effect of information bias introduced by selecting cases from EUROCAT NNL and controls from the Lifelines cohort. An exposure–response analysis was conducted for maternal occupational exposure and CHD in general. If <5 infants were exposed, data was not presented and OR were not estimated.

**Table 1:** Baseline characteristics of Lifelines controls and Eurocat cases. [CHD=congenital heart defects.]

	Controls (N=5602)		CHD (N=1174)		P-value
	
	N	%	N	%	
Child sex					<0.01
Male	2731	48.8	632	53.8	
Female	2871	51.2	542	46.2	
Birth year					0.12
1997–2000	1240	22.1	266	22.8	
2001–2004	1660	29.6	310	26.1	
2005–2008	1293	23.1	298	25.5	
2009–2013	1409	25.2	300	25.6	
Maternal age at delivery					<0.01
15–19 ^a^	3	0.1	1	0.1	
20–24	191	3.6	100	8.7	
25–29	1492	28.2	362	31.6	
30–34	2470	46.7	479	16.2	
35–39	1058	20.0	194	16.9	
≥40	73	1.4	11	1.0	
Unknown	315		27		
Education level					<0.01
Low	649	12.3	162	14.0	
Middle	2396	45.4	561	48.6	
High	2236	42.3	432	37.4	
Unknown	321		19		
Body mass index (kg/m^2^) ^b^					<0.01
<18.5	56	1.0	31	2.7	
18.5–24.9	2871	53.6	738	64.6	
25.0–29.9	1610	30.1	269	23.5	
≥30	818	15.3	105	9.2	
Unknown	247		32		
Smoking during first trimester					<0.01
No	5036	90.2	903	77.2	
Yes	549	9.8	267	22.8	
Unknown	17		4		
Alcohol during first trimester					<0.01
No	5045	90.3	873	74.6	
Yes	544	9.7	297	25.4	
Unknown	13		4		
Folic acid use					<0.01
No	847	16.5	290	24.9	
Yes	4272	83.5	873	75.1	
Unknown	483		11		
Fertility problems					<0.01
No	5230	93.9	971	83.6	
Yes	339	6.1	190	16.4	
Unknown	33		13		

a Lifelines includes participants from 18 years old.

b Body mass index of Eurocat cases is based on self-reported height and weight. Height and weight of Lifelines participants is measured at the baseline visit to the study clinic.

## Results

Baseline characteristics differed between cases and controls (table 1). Infants born with a CHD were more often boys. Mothers of case infants had a lower maternal age at delivery, lower educational level, and lower BMI. As expected, they were also more likely to smoke or consume alcohol, used folic acid supplements less often, and had more fertility problems compared to mothers of controls.

In total, 37.6% of CHD infants and 35.6% of the control infants were exposed to any of the maternal occupational exposures early in pregnancy (table 2), and no association was found between any exposure and CHD in general. When examining any exposure and specific groups of CHD, we found an association for pulmonary (valve) stenosis in combination with ventricular septal defect (VSD) (OR_adj_ 3.06, 95% CI 1.20–7.81). However, any exposure is also associated with a lower risk of aortic stenosis (OR_adj_ 0.32, 95% CI 0.11–0.94).

**Table 2a:** Prevalence, crude and adjusted odds ratio (OR_crude_/OR_adj_) of maternal occupational exposure and congenital heart defects (CHD) in the offspring. [CI=confidence interval; d-TGA=dextro-transposition of the great arteries; LVOTO/RVOTO=left/right ventricular outflow tract obstruction; HLHS=hypoplastic left heart syndrome; P(v)S=pulmonary (valve) stenosis; CoA=coarctation of aorta; VSD=ventricular septal defect; ASD=atrial septal defect; AVSD=atrioventricular septal defect; APVR=anomalous pulmonary venous return; NC=not calculated due to sparse data]. BOLD indicates significant values (P<0.05).

CHD classification	Total	Any occupational exposure						Organic dust exposure					
	
		N	%	OR_crude_	95% CI	OR_adj_^a^	95% CI	N	%	OR_crude_	95% CI	OR_adj_^a^	95% CI
Controls	5602	1992	35.6	Ref		Ref		1617	28.9	Ref		Ref	
Total CHD	1174	442	37.6	1.09	0.96–1.25	1.04	0.90–1.20	356	30.3	1.07	0.94–1.23	1.10	0.95–1.28
Conotruncal	174	69	39.7	1.19	0.88–1.62	1.13	0.81–1.57	57	32.8	1.20	0.87–1.66	1.30	0.93–1.82
d-TGA	74	28	37.8	1.10	0.69–1.77	1.00	0.60–1.68	23	31.1	1.11	0.68–1.82	1.18	0.70–1.99
Tetralogy of Fallot	60	28	46.7	1.59	0.95–2.64	1.50	0.88–2.57	23	38.3	1.53	0.91–2.59	1.68	0.98–2.89
Truncus arteriosus	10	5	50.0	1.81	0.52–6.27	1.46	0.41–5.24	<5		NC		NC	
LVOTO	173	62	35.8	1.01	0.74–1.39	0.94	0.67–1.31	53	30.6	1.09	0.78–1.51	1.14	0.80–1.60
HLHS	50	19	38.0	1.11	0.63–1.97	0.86	0.47–1.57	15	30.0	1.06	0.58–1.94	0.87	0.46–1.67
Aortic stenosis	31	5	16.1	0.35	0.13–0.91	0.32	0.11–0.94	<5		NC		NC	
Coarctation of aorta	42	18	42.9	1.36	0.74–2.51	1.33	0.70–2.54	17	40.5	1.68	0.90–3.11	1.90	1.01–3.59
Bicuspid aortic valve	42	16	38.1	1.12	0.60–2.08	1.14	0.59–2.18	14	33.3	1.23	0.65–2.35	1.37	0.70–2.65
RVOTO	139	58	41.7	1.30	0.92–1.83	1.24	0.87–1.77	49	35.3	1.34	0.94–1.91	1.35	0.94–1.95
P(v)S	104	44	42.3	1.33	0.90–1.97	1.26	0.84–1.90	37	35.6	1.36	0.91–2.04	1.36	0.89–2.07
Pulmonary atresia	13	6	46.2	1.55	0.52–4.63	1.37	0.45–4.20	6	46.2	2.11	0.71–6.30	2.13	0.70–6.43
Septal	544	194	35.7	1.01	0.84–1.21	0.96	0.79–1.17	150	27.6	0.94	0.77–1.14	0.97	0.79–1.19
Perimembranous VSD	117	51	43.6	1.40	0.97–2.03	1.34	0.91–1.99	40	34.2	1.28	0.87–1.88	1.40	0.94–2.10
Muscular VSD	248	79	31.9	0.85	0.65–1.11	0.87	0.65–1.16	63	25.4	0.84	0.63–1.12	0.90	0.67–1.22
Other VSD	78	27	34.6	0.96	0.60–1.53	0.98	0.60–1.61	18	23.1	0.74	0.44–1.26	0.82	0.47–1.41
ASD	98	36	36.7	1.05	0.70–1.59	0.93	0.60–1.43	28	28.6	0.99	0.63–1.53	0.95	0.60–1.51
AVSD	28	7	25.0	0.60	0.26–1.42	0.67	0.27–1.63	6	21.4	0.67	0.27–1.66	0.81	0.32–2.06
APVR	17	9	52.9	2.04	0.79–5.29	1.88	0.69–5.10	8	47.1	2.19	0.84–5.69	1.99	0.73–5.41
Total APVR	11	5	45.5	1.51	0.46–4.96	1.48	0.44–4.97	5	45.5	2.05	0.63–6.74	2.03	0.61–6.76
Complex	45	19	42.2	1.32	0.73–2.40	1.30	0.68–2.47	16	35.6	1.36	0.74–2.51	1.39	0.72–2.70
Single ventricle	14	8	57.1	2.42	0.84–6.97	2.54	0.80–8.11	6	42.9	1.85	0.64–5.34	2.13	0.69–6.54
Associations													
CoA + VSD	15	7	46.7	1.59	0.57–4.38	1.69	0.59–4.83	5	33.3	1.23	0.42–3.61	1.36	0.56–4.08
P(v)S + VSD	19	11	57.9	**2.49**	**1.00–6.21**	**3.06**	**1.20–7.81**	**9**	47.4	2.22	0.90–5.47	**2.68**	**1.07–6.73**

a Adjusted for child sex, maternal age at delivery (as continuous variable), education level, maternal BMI (as continuous variable), smoking and alcohol use during pregnancy, folic acid supplementation, and fertility problems.

**Table 2b:** Prevalence, crude and adjusted odds ratio (OR_crude_/OR_adj_) of maternal occupational exposure and congenital heart defects (CHD) in the offspring. [CI=confidence interval; d-TGA=dextro-transposition of the great arteries; LVOTO/RVOTO=left/right ventricular outflow tract obstruction; HLHS=hypoplastic left heart syndrome; P(v)S=pulmonary (valve) stenosis; CoA=coarctation of aorta; VSD=ventricular septal defect; ASD=atrial septal defect; AVSD=atrioventricular septal defect; APVR=anomalous pulmonary venous return; NC=not calculated due to sparse data]. BOLD indicates significant values (P<0.05).

CHD classification	Total	Mineral dust exposure						Solvents exposure					
	
		N	%	OR_crude_	95% CI	OR_adj_^a^	95% CI	N	%	OR_crude_	95% CI	OR_adj_^a^	95% CI
Controls	5602	418	7.5	Ref		Ref		1370	24.5	Ref		Ref	
Total CHD	1174	120	10.2	**1.41**	**1.14–1.75**	**1.29**	**1.01–1.64**	275	23.4	0.95	0.82–1.10	0.95	0.81–1.11
Conotruncal	174	18	10.3	1.43	0.87–2.36	1.31	0.76–2.26	40	23.0	0.92	0.64–1.32	1.00	0.69–1.45
d-TGA	74	6	8.1	1.09	0.47–2.54	0.95	0.37–2.46	16	21.6	0.85	0.49–1.49	0.98	0.55–1.73
Tetralogy of Fallot	60	8	13.3	1.91	0.90–4.04	1.77	0.80–3.94	18	30.0	1.32	0.76–2.31	1.38	0.78–2.43
Truncus arteriosus	10	<5		NC		NC		<5		NC		NC	
LVOTO	173	22	12.7	**1.81**	**1.14–2.86**	**1.75**	**1.06–2.89**	37	21.4	0.84	0.58–1.22	0.81	0.55–1.20
HLHS	50	7	14.0	2.02	0.90–4.52	1.51	0.62–3.72	11	22.0	0.87	0.45–1.71	0.75	0.37–1.52
Aortic stenosis	31	<5		NC		NC		<5		NC		NC	
Coarctation of aorta	42	7	16.7	**2.48**	**1.10–5.62**	**2.94**	**1.21–7.13**	8	19.0	0.73	0.34–1.57	0.74	0.34–1.63
Bicuspid aortic valve	42	5	11.9	1.68	0.66–4.29	1.56	0.58–4.18	11	26.2	1.10	0.55–2.19	1.18	0.58–2.40
RVOTO	139	19	13.7	**1.96**	**1.20–3.22** ^b^	**1.75**	**1.02–3.00**	37	26.6	1.12	0.77–1.64	1.13	0.76–1.66
P(v)S	104	16	15.2	**2.26**	**1.31–3.88**^b^	**1.99**	**1.10–3.62**	26	25.0	1.03	0.66–1.61	1.05	0.66–1.65
Pulmonary atresia	13	<5		NC		NC		<5		NC		NC	
Septal	544	48	8.8	1.20	0.88–1.64	1.06	0.75–1.49	121	22.2	0.88	0.72–1.09	0.90	0.72–1.13
Perimembranous VSD	117	12	10.3	1.42	0.77–2.60	1.31	0.69–2.50	32	27.4	1.16	0.77–1.75	1.24	0.81–1.89
Muscular VSD	248	21	8.5	1.15	0.73–1.82	1.15	0.71–1.88	52	21.0	0.82	0.60–1.12	0.84	0.61–1.16
Other VSD	78	7	9.0	1.22	0.56–2.68	1.11	0.48–2.53	12	15.4	0.56	0.30–1.04	0.60	0.32–1.12
ASD	98	8	8.2	1.10	0.53–2.29	0.83	0.38–1.80	24	24.5	1.00	0.63–1.59	0.97	0.60–1.58
AVSD	28	<5		NC		NC		<5		NC		NC	
APVR	17	<5		NC		NC		8	47.1	**2.75**	**1.06–7.13**	2.18	0.80–5.93
Total APVR	11	<5		NC		NC		5	45.5	2.57	0.78–8.45	2.23	0.67–7.42
Complex	45	<5		NC		NC		15	33.3	1.55	0.83–2.88	1.58	0.82–3.07
Single ventricle	14	<5		NC		NC		5	35.7	1.72	0.57–5.13	1.97	0.62–6.20
Associations													
CoA + VSD	15	<5		NC		NC		<5		NC		NC	
P(v)S + VSD	19	<5		NC		NC		7	36.8	1.80	0.71–4.59	1.82	0.70–4.73

a Adjusted for child sex, maternal age at delivery (as continuous variable), education level, maternal BMI (as continuous variable), smoking and alcohol use during pregnancy, folic acid supplementation, and fertility problems.

b P-value <0.01.

**Table 2c:** Prevalence, crude and adjusted odds ratio (OR_crude_/OR_adj_) of maternal occupational exposure and congenital heart defects (CHD) in the offspring. [CI=confidence interval; d-TGA=dextro-transposition of the great arteries; LVOTO/RVOTO=left/right ventricular outflow tract obstruction; HLHS=hypoplastic left heart syndrome; P(v)S=pulmonary (valve) stenosis; CoA=coarctation of aorta; VSD=ventricular septal defect; ASD=atrial septal defect; AVSD=atrioventricular septal defect; APVR=anomalous pulmonary venous return; NC=not calculated due to sparse data]. BOLD indicates significant values (P<0.05).

CHD classification	Total	Pesticides exposure						Metal dust and fumes exposure					
	
		N	%	OR_crude_	95% CI	OR_adj_^a^	95% CI	N	%	OR_crude_	95% CI	OR_adj_^a^	95% CI
Controls	5602	131	2.3	Ref		Ref		20	0.4	Ref		Ref	
Total CHD	1174	34	2.9	1.25	0.85–1.83	1.20	0.79–1.81	12	1.0	**2.88**	**1.41–5.91**	**2.40**	**1.09–5.30**
Conotruncal	174	<5		NC		NC		<5		NC		NC	
d-TGA	74	<5		NC		NC		<5		NC		NC	
Tetralogy of Fallot	60	<5		NC		NC		<5		NC		NC	
Truncus arteriosus	10	<5		NC		NC		<5		NC		NC	
LVOTO	173	7	4.0	1.76	0.81–3.83	1.73	0.77–3.87	<5		NC		NC	
HLHS	50	<5		NC		NC		<5		NC		NC	
Aortic stenosis	31	<5		NC		NC		<5		NC		NC	
Coarctation of aorta	42	<5		NC		NC		<5		NC		NC	
Bicuspid aortic valve	42	<5		NC		NC		<5		NC		NC	
RVOTO	139	5	3.6	1.56	0.63–3.87	1.45	0.57–3.67	<5		NC		NC	
P(v)S	104	<5		NC		NC		<5		NC		NC	
Pulmonary atresia	13	<5		NC		NC		<5		NC		NC	
Septal	544	16	2.9	1.27	0.75–2.14	1.20	0.69–2.09	6	1.1	**3.11**	**1.25–7.78**	2.47	0.92–6.64
Perimembranous VSD	117	5	4.3	1.86	0.75–4.64	1.80	0.70–4.61	<5		NC		NC	
Muscular VSD	248	5	2.0	0.86	0.35–2.12	0.87	0.35–2.20	<5		NC		NC	
Other VSD	78	<5		NC		NC		<5		NC		NC	
ASD	98	<5		NC		NC		<5		NC		NC	
AVSD	28	<5		NC		NC		<5		NC		NC	
APVR	17	<5		NC		NC		<5		NC		NC	
Total APVR	11	<5		NC		NC		<5		NC		NC	
Complex	45	<5		NC		NC		<5		NC		NC	
Single ventricle	14	<5		NC		NC		<5		NC		NC	
Associations													
CoA + VSD	15	<5		NC		NC		<5		NC		NC	
P(v)S + VSD	19	<5		NC		NC		<5		NC		NC	

a Adjusted for child sex, maternal age at delivery (as continuous variable), education level, maternal BMI (as continuous variable), smoking and alcohol use during pregnancy, folic acid supplementation, and fertility problems.

When analyzing specific exposures, the most prevalent maternal occupational exposure was to organic dust, with approximately 30% of women exposed. Associations were found between organic dust exposure and coarctation of aorta (OR_adj_ 1.90, 95% CI 1.01–3.59) and pulmonary (valve) stenosis in combination with VSD (OR_adj_ 2.68, 95% CI 1.07–6.73). Mineral dust exposure was less common (10% of cases and 8% of controls) and was associated with CHD in general (OR_adj_ 1.29, 95% CI 1.01–1.64). When analyzing mineral dust exposure in relation to specific CHD, we found an association with LVOTO defects (OR_adj_ 1.75, 95% CI 1.06–2.89), particularly coarctation of the aorta (OR_adj_ 2.94, 95% CI 1.21–7.13), and with RVOTO defects, especially pulmonary (valve) stenosis (OR_adj_ 1.99, 95% CI 1.10–3.62). Approximately 25% of mothers were exposed to solvents and 2–3% to pesticides, but no associations between exposure to solvents or pesticides and CHD were found. Although the prevalence of exposure to metal dust and fumes was only 0.4% for controls and 1% for cases, we did observe an association between this exposure and CHD in general (OR_adj_ 2.40, 95% CI 1.09–5.30). When mothers were exposed to mineral dust and metal dust and fumes, the association with CHD in general became stronger compared to exposure to mineral dust or metal dust and fumes alone (OR_adj_ 2.92, 95% CI 1.23–6.92), and an association with septal defects was found (OR_adj_ 3.23, 95% CI 1.14–9.11) (supplementary material, www.sjweh.fi/show_abstract.php?abstract_id=3912, table S1).

Stratified analysis by isolated and multiple defects included 1009 cases with isolated CHD and 165 cases with CHD and extra-cardiac malformations. The OR_adj_ for isolated CHD were comparable to the total group of CHD (supplementary table S2). One additional association was observed when only isolated defects were included: exposure to metal dust and fumes was associated with septal defects (OR_adj_ 3.06, 95% CI 1.14–8.23). The OR_adj_ for multiple defects that include CHD showed no association for any of the exposures (supplementary table S3). Only a small number of cases were included in the stratified analyses for multiple defects, and most OR_adj_ were not estimated due to sparse outcome and exposure data.

The analyses restricted to non-smoking mothers who did not report drinking alcohol early in pregnancy to explore the effect of information bias included 703 cases and 4622 controls (supplementary table S4), and the association between maternal occupational exposure to mineral dust and LVOTO anomalies was not observed. Due to few data it was not possible to explore specific subgroups of LVOTO anomalies in this sensitivity analysis.

An exposure–response analysis was performed for any exposure and CHD in general. The OR_adj_ appeared to be non-significant but higher in the high exposure group only (OR_adj_ 1.37, 95% CI 0.97–1.94; supplementary table S5).

## Discussion

This study showed that infants with specific CHD were more likely to be exposed in utero to organic and mineral dust and metal dust and fumes at the workplace of mother compared with infants without malformations. Exposure to organic dust was associated with a two-fold increased risk of coarctation of aorta and a three-fold increased risk of pulmonary (valve) stenosis in combination with VSD. Organic dust includes exposure to smaller particles such as fungal and bacterial spores/cells, pollen, viruses, or fragments of larger organisms including cotton and wood dust, flour, textile and paper fibers. All mothers exposed to organic dust were considered to be relatively low exposed. Almost two thirds of these women worked in health or personal care, another 15% worked as cleaners and 13% worked in agriculture or the food industry. Mineral dust exposure was associated with a two-fold increase in LVOTO defects in offspring, specifically coarctation of the aorta. Exposure to mineral dust exposure was also associated with a two-fold risk of RVOTO defects, specifically pulmonary (valve) stenosis. Mineral dusts are aerosols originating from minerals, such as from the soil, (non-fibrous) silica dusts, and coal. Of the 12% of women high exposed to mineral dust, 90% were working in agriculture/horticulture. Of the low-exposed women, 50% worked as cleaners and the rest in various jobs such as metal, electronics, plastics production and dairy and livestock production. Exposure to metal dust and fumes was associated with a two-fold increase of CHD in general. However, this result has to be interpreted carefully as only 1% of the women, mostly those working as machine and instrument operators/repairers, were occupationally exposed to metal dust and fumes. Exposure to mineral dust in combination with metal dust and fumes was associated with a three-fold increased risk of septal defects. We also found that infants affected by aortic stenosis were less likely to be exposed to any maternal occupational exposure compared to non-malformed controls. However, only five cases with aortic stenosis were included, and analyses for specific subgroups of exposure could not be performed. No specific job association was identified.

During their work, mothers may inhale mineral, metal or organic aerosols, which can pass through the lungs into the blood. These agents might consequently cross the placental barrier and have been found at the foetal side of the placenta (24). Occupational exposures – including to several organic, mineral, and metal compounds– can induce oxidative stress, which may induce teratogenesis via misregulation of critical pathways involved in foetal development (25).

Although the association between metal dust and fumes and CHD/isolated septal defects has to be interpreted with caution, previous studies have found increased risks. One study found an association between exposure to metals and specific septal defects (26). Two other studies showed that maternal occupational exposure to mineral oils, which are often used in the metal industry, increased the risk of isolated septal defects (27) and coarctation of the aorta (28). Another study using comparable methods did not show this association, but these estimates could have been imprecise as this study included less than five exposed cases (28). To our knowledge, no studies specifically examining organic or mineral dust have been reported.

Our results did not confirm the association between occupational exposure to solvents and CHD reported by a meta-analysis using similar occupational exposure assessment methods (14). It is possible that the difference is explained by the diversity of CHD included in the meta-analysis. One previous study assessing solvent exposure and specific types of CHD also showed no association (29), but another study found an association with peri membranous VSD and aorta stenosis (30). Our results on maternal occupational exposure to pesticides are in line with the meta-analysis, which also found no association with CHD (14). One previous study found an association between pesticide exposure and specific CHD, such as RVOTO defects, hypoplastic left heart syndrome, and tetralogy of Fallot (31). Unfortunately, our sample size was too limited to analyze these specific CHD.

### Limitations

Occupational exposure assessment using the ALOHA+ JEM is done at job level, which could have resulted in misclassification of exposure. Circumstances at the workplace are often unpredictable and can vary within jobs, between workplaces and over time. It is also possible that women avoided certain exposures because they wanted to become pregnant or knew they were pregnant while performing a job that would normally come with these exposures. The limited number of exposed women could have resulted in high OR with large CI and restricted our ability to explore exposure–response associations.

Because EUROCAT NNL does not collect data on non-malformed controls, controls were selected from Lifelines, and this approach introduced several limitations. EUROCAT NNL aims to investigate the prevalence and risk factors for congenital anomalies, and its questionnaire is focused specifically on risk factors for congenital anomalies. Lifelines collects data to obtain insight into healthy ageing, and specifically for children on neonatal and childhood diseases. Consequently, the Lifelines questionnaire includes items on a wide variety of risk factors. These differences could introduce information bias during assessment of the covariates. We assume that bias was not introduced for maternal occupational exposure as mothers were asked to report a description of their job early in pregnancy in both questionnaires, and recall bias is limited for self-reported jobs (32). After exploration of the effect of information bias in a sensitivity analysis, it seems that the effect of information bias is limited as only the association between maternal occupational exposure to mineral dust and LVOTO anomalies was no longer observed. Additionally, residual confounding due to maternal diabetes, paternal smoking, environmental exposures, or other occupational factors not covered by the ALOHA+ JEM could have been introduced since information regarding those risk factors was lacking.

Another major concern of using Lifelines is selection bias. Previous studies showed that some groups of individuals, for example those with a low socioeconomic status, are less likely to participate in population-based cohort studies (33, 34). However, Lifelines is known to be representative of the population in the northern Netherlands, indicating selection bias might be low (35).

### Strengths

A major strength of this study is the high quality of data from EUROCAT NNL, which registers detailed medical information for each case. Anomalies were coded by trained registry staff according to international coding guidelines (18). Case classification was performed under supervision of an experienced clinical geneticist and a pediatric cardiologist. Use of the Botto classification made it possible to create homogenous groups of CHD based on anatomy and developmental and epidemiological evidence (19). Another strength is that EUROCAT identifies eligible cases by active case ascertainment using various sources in the catchment area, and ~72% of the parents of a child affected by a CHD agreed to participate and responded to the questionnaire. A major strength of the JEM approach is that it limits the effect of recall bias on exposure status as well as differential misclassification of exposure when compared to self-reported exposure (22, 36).

### Concluding remarks

This large population-based case–control study showed that maternal occupational exposure to organic dust, mineral dust, and metal dust and fumes early in pregnancy could possibly affect the development of the foetal heart. These exposures, with a prevalence of 1–30% at the workplace, were associated with a two- to three-fold increase in LVOTO, RVOTO, and septal defects in this study. Despite the limitations of this study, women should be careful if they are exposed at work to mineral and organic dusts and metal dust and fumes in the months before and early in pregnancy.

## Supplementary material

Supplementary material

## Protection of research participants

EUROCAT NNL and Lifelines operate within the scope of the Dutch Data Protection Act, according to the principles of the Declaration of Helsinki and in accordance with the research code of the UMCG, The Netherlands, and the Code of Good conduct. EUROCAT NNL did not require ethics committee approval to collect and store data according to the medical ethical committee of the UMCG in line with the act Medical Research involving Human Subjects. The medical ethical committee of the UMCG approved Lifelines. All parents and participants of EUROCAT NNL and Lifelines give informed consent before participation.
