# Supplementary material for: Maternal occupational exposure and congenital heart defects in offspring
Source: Scand J Work Environ Health. 2020 Oct 30;46(6):599–608. doi: 10.5271/sjweh.3912 (PMC7737813; doi:10.5271/sjweh.3912)
Supplement: Supplementary material [file SJWEH-46-599-S001.pdf]

Maternal occupational exposure and congenital heart defects in offspring[1]

by Nynke Spinder, MD,[2] Jorieke EH Bergman, MD, PhD, Hans Kromhout, PhD, Roel Vermeulen, PhD, Nicole Corsten-Janssen, MD, H Marike Boezen, PhD, Gideon J du Marchie Sarvaas, MD, Hermien EK de Walle, PhD

1. Supplementary *tables*
2. *Correspondence to: Nynke Spinder, Department of Epidemiology, University of Groningen, University Medical Center Groningen, Hanzeplein 1, P.O. Box 30.001, 9700 RB Groningen, The Netherlands. [E-mail: n.spinder@umcg.nl*]

Table S1 Prevalence, crude OR and adjusted OR of maternal occupational exposures and CHDs in offspring.

	Controls		CHDs		Unadjusted		Adjusted [a]	
	n	(%)	n	(%)	OR	95% CI	OR	95% CI
Any CHD	(n=5602)		(n=1174)					
No exposure	5179	(92.4%)	1053	(89.7%)	Ref		Ref	
Mineral dust only	403	(7.2%)	109	(9.3%)	1.33	1.07-1.66	1.22	0.95-1.57
Metal dust and fumes only	5	(0.1%)	<5		NC		NC	
Both	15	(0.3%)	11	(0.9%)	**3.61**	**1.65-7.88**	**2.92**	**1.23-6.92**
Septal defects	(n=5602)		(n=544)					
No exposure	5179	(92.4%)	496	(91.2%)	Ref		Ref	
Mineral dust only	403	(7.2%)	42	(7.7%)	1.09	0.78-1.52	0.96	0.67-1.38
Metal dust and fumes only	13	(1.1%)	<5		NC		NC	
Both	15	(0.3%)	6	(1.1%)	**4.18**	**1.61-10.81**	**3.23**	**1.14-9.11**

CHD, congenital heart defects; LVOTO, left ventricular outflow tract obstruction; NC, not calculated due to sparse data. [a] Adjusted for child sex, maternal age at delivery (as continuous variable), educational level, maternal BMI (as continuous variable), smoking and alcohol use during pregnancy, folic acid supplementation, and fertility problems. **Bold indicates significant value (P<0.05)**

Table S2 Prevalence, crude OR and adjusted OR of maternal periconceptional exposure and isolated CHDs in the offspring.

		Occupational exposure												
		Any exposure						Organic dust						
	Total	Exposed		Unadjusted		Adjusted [a]		Exposed		Unadjusted		Adjusted [a]		
CHD classification	n	n	%	OR	95% CI	OR	95% CI	n	%	OR	95% CI	OR	95% CI	
Controls	5602	1992	(35.6%)	Ref		Ref	1992	1617	(28.9%)	Ref		Ref		
Total CHD	1009	374	(37.1%)	1.07	0.93-1.23	1.02	0.871.19	300	(29.7%)	1.04	0.90-1.21	1.07	0.91-1.26	
Conotruncal	144	55	(38.2%)	1.12	0.80-1.57	1.08	0.75-1.55	45	(31.3%)	1.12	0.78-1.60	1.21	0.84-1.76	
d-TGA	64	23	(35.9%)	1.02	0.61-1.70	0.95	0.54-1.65	18	(28.1%)	0.96	0.56-1.67	1.06	0.60-1.89	
Tetralogy of Fallot	45	21	(46.7%)	1.59	0.88-2.86	1.56	0.85-2.88	17	(37.8%)	1.50	0.82-2.74	1.60	0.85-2.96	
LVOTO	155	55	(35.5%)	1.00	0.72-1.39	0.92	0.64-1.32	46	(29.7%)	1.04	0.73-1.48	1.09	0.75-1.58	
HLHS	45	17	(37.8%)	1.10	0.60-2.02	0.83	0.44-1.59	13	(28.9%)	1.00	0.52-1.91	0.80	0.40-1.61	
Aortic stenosis	28	5	(17.9%)	0.40	0.15-1.04	0.37	0.13-1.10	<5		NC		NC		
Coarctation of aorta	39	16	(41.0%)	1.26	0.66-2.39	1.19	0.61-2.34	15	(38.5%)	1.54	0.81-2.94	1.73	0.89-3.37	
Bicuspid aortic valve	37	15	(40.5%)	1.24	0.64-2.39	1.25	0.63-2.49	13	(35.1%)	1.34	0.68-2.63	1.49	0.74-2.99	
RVOTO	127	52	(40.9%)	1.26	0.88-1.80	1.16	0.80-1.68	43	(33.9%)	1.26	0.87-1.83	1.24	0.84-1.82	
P(v)S	94	56	(40.4%)	1.23	0.81-1.86	1.12	0.73-1.73	31	(33.0%)	1.21	0.79-1.87	1.18	0.75-1.84	
Pulmonary atresia	13	6	(46.2%)	1.55	0.52-4.63	1.37	0.45-4.20	6	(46.2%)	2.11	0.71-6.30	2.13	0.70-6.43	
Septal	459	164	(35.7%)	1.01	0.83-1.23	0.99	0.80-1.22	129	(28.1%)	0.96	0.78-1.19	1.01	0.80-1.26	
Perimembranous VSD	91	41	(45.1%)	1.49	0.98-2.25	1.50	0.97-2.32	33	(36.3%)	1.40	0.91-2.16	1.54	0.99-2.40	
Muscular VSD	218	70	(32.1%)	0.86	0.64-1.15	0.88	0.65-1.20	57	(26.1%)	0.87	0.64-1.19	0.94	0.68-1.29	
Other VSD	67	22	(32.8%)	0.89	0.53-1.48	0.95	0.56-1.63	14	(20.9%)	0.65	0.36-1.18	0.76	0.41-1.40	
ASD	82	31	(37.8%)	1.10	0.70-1.73	0.97	0.60-1.55	25	(30.5%)	1.08	0.67-1.74	1.03	0.63-1.68	
AVSD	24	5	(20.8%)	0.48	0.18-1.28	0.51	0.18-1.42	<5		NC		NC		
APVR	15	8	(53.3%)	2.07	0.75-5.72	1.83	0.63-5.34	7	(46.7%)	2.16	0.78-5.96	1.87	0.64-5.45	
Total APVR	11	5	(45.5%)	1.51	0.46-4.96	1.48	0.44-4.97	5	(45.5%)	2.05	0.63-6.74	2.03	0.61-6.76	
Complex	38	16	(42.1%)	1.32	0.69-2.52	1.18	0.58-2.37	13	(34.2%)	1.28	0.65-2.51	1.22	0.59-2.53	
Single ventricle	11	7	(63.6%)	3.17	0.93-10.85	2.56	0.72-9.09	5	(45.5%)	2.05	0.63-6.74	1.96	0.58-6.64	
Associations														
CoA + VSD	14	7	(50.0%)	1.81	0.64-5.17	1.88	0.63-5.58	5	(35.7%)	1.37	0.46-4.09	1.50	0.49-4.59	
P(v)S + VSD	17	9	(52.9%)	2.04	0.79-5.29	2.46	0.92-6.57	7	(41.2%)	1.73	0.66-4.54	2.07	0.77-5.56	

CHD, congenital heart defects; d-TGA, dextro-transposition of the great arteries; LVOTO, left ventricular outflow tract obstruction; HLHS, hypoplastic left heart syndrome; RVOTO, right ventricular outflow tract obstruction; P(v)S, pulmonary (valve) stenosis; CoA, coarctation of aorta; VSD, ventricular septal defect; ASD, atrial septal defect; AVSD, atrioventricular septal defect; APVR, anomalous pulmonary venous return; NC, not calculated due to sparse data. [a] Adjusted for child sex, maternal age at delivery (as continuous variable), education level, maternal BMI (as continuous variable), smoking and alcohol use during pregnancy, folic acid supplementation, and fertility problems. *p-value <0.01.

Table S2 Continued.

		Occupational exposure											
		Mineral dust						Solvents					
	Total	Exposed		Unadjusted		Adjusted [a]		Exposed		Unadjusted		Adjusted [a]	
CHD classification	n	n	%	OR	95% CI	OR	95% CI	n	%	OR	95% CI	OR	95% CI
Controls	5602	418	(7.5%)	Ref		Ref		1370	(24.5%)	Ref		Ref	
Total CHD	1174	104	(10.3%)	**1.43**	**1.14-1.79**	**1.33**	**1.03-1.72**	238	(23.6%)	0.95	0.82-1.12	0.95	0.80-1.13
Conotruncal	144	13	(9.0%)	1.23	0.69-2.20	1.18	0.64-2.18	35	(24.3%)	0.99	0.68-1.46	1.07	0.72-1.58
d-TGA	64	<5		NC		NC		15	(23.4%)	0.95	0.53-1.67	1.10	0.60-1.99
Tetralogy of Fallot	45	5	(11.1%)	1.55	0.61-3.95	1.43	0.53-3.82	15	(33.3%)	1.55	0.83-2.88	1.55	0.82-2.93
LVOTO	155	21	(13.5%)	**1.94**	**1.21-3.11***	**1.93**	**1.15-3.23**	32	(20.6%)	0.80	0.54-1.19	0.77	0.51-1.17
HLHS	45	7	(15.6%)	**2.29**	**1.01-5.15**	1.82	0.74-4.51	9	(20.0%)	0.77	0.37-1.61	0.63	0.29-1.37
Aortic stenosis	28	<5		NC		NC		<5		NC		NC	
Coarctation of aorta	39	7	(17.9%)	**2.71**	**1.19-6.18**	**3.12**	**1.27-7.65**	7	(17.9%)	0.68	0.30-1.53	0.69	0.30-1.58
Bicuspid aortic valve	37	5	(13.5%)	1.94	0.75-5.00	1.73	0.64-4.69	10	(27.0%)	1.14	0.55-2.37	1.25	0.59-2.65
RVOTO	127	17	(13.4%)	**1.92**	**1.14-3.23**	**1.61**	**0.91-2.84**	33	(26.0%)	1.08	0.73-1.62	1.08	0.72-1.63
P(v)S	94	14	(14.9%)	**2.17**	**1.22-3.86***	1.79	0.95-3.37	22	(23.4%)	0.94	0.58-1.53	0.95	0.58-1.55
Pulmonary atresia	13	<5		NC		NC		<5		NC		NC	
Septal	459	41	(8.9%)	1.22	0.87-1.70	1.12	0.78-1.62	107	(23.3%)	0.94	0.75-1.18	0.96	0.75-1.21
Perimembranous VSD	91	10	(11.0%)	1.53	0.79-2.98	1.47	0.73-2.98	27	(29.7%)	1.30	0.83-2.05	1.38	0.87-2.20
Muscular VSD	218	19	(8.7%)	1.18	0.73-1.92	1.23	0.74-2.06	48	(22.0%)	0.87	0.63-1.21	0.89	0.63-1.24
Other VSD	67	5	(7.5%)	1.00	0.40-2.50	1.02	0.39-2.66	12	(17.9%)	0.67	0.36-1.26	0.72	0.38-1.37
ASD	82	7	(8.5%)	1.16	0.53-2.53	0.88	0.38-2.00	20	(24.4%)	1.00	0.60-1.66	0.95	0.56-1.62
AVSD	24	<5		NC		NC			NC		NC		
APVR	17	<5		NC		NC		7	(46.7%)	2.70	0.98-7.47	2.07	0.71-6.03
Total APVR	11	<5		NC		NC		5	(45.5%)	2.57	0.78-8.45	2.23	0.67-7.42
Complex	38	<5		NC		NC							
Single ventricle	11	<5		NC		NC		12	(31.6%)	1.43	0.72-2.83	1.41	0.68-2.92
Associations								<5		NC		NC	
CoA + VSD	14	<5		NC		NC		<5		NC		NC	
P(v)S + VSD	17	<5		NC		NC		6	(35.3%)	1.69	0.62-4.57	1.71	0.62-4.74

CHD, congenital heart defects; d-TGA, dextro-transposition of the great arteries; LVOTO, left ventricular outflow tract obstruction; HLHS, hypoplastic left heart syndrome; RVOTO, right ventricular outflow tract obstruction; P(v)S, pulmonary (valve) stenosis; CoA, coarctation of aorta; VSD, ventricular septal defect; ASD, atrial septal defect; AVSD, atrioventricular septal defect; APVR, anomalous pulmonary venous return; NC, not calculated due to sparse data. [a] Adjusted for child sex, maternal age at delivery (as continuous variable), education level, maternal BMI (as continuous variable), smoking and alcohol use during pregnancy, folic acid supplementation, and fertility problems. *p-value <0.01. **Bold indicates significant value (P<0.05)**

Table S2 Continued.

		Occupational exposure													
		Pesticides						Metal dust and fumes							
	Total	Exposed		Unadjusted		Adjusted [a]		Exposed		Unadjusted		Adjusted [a]			
CHD classification	n	n	%	OR	95% CI	OR	95% CI	n	%	OR	95% CI	OR	95% CI		
Controls	5602	131	(2.3%)	Ref		Ref		20	(0.4%)	Ref		Ref			
Total CHD	1174	29	(2.9%)	1.24	0.82-1.24	1.20	0.77-1.86	12	(1.2%)	**3.36**	**1.64-6.90***	**2.81**	**1.28-6.16**		
Conotruncal	144	<5		NC		NC		<5		NC		NC			
d-TGA	64	<5		NC		NC		<5		NC		NC			
Tetralogy of Fallot	45	<5		NC		NC		<5		NC		NC			
LVOTO	155	7	(4.5%)	1.98	0.91-4.30	1.98	0.88-4.46	<5		NC		NC			
HLHS	45	<5		NC		NC		<5		NC		NC			
Aortic stenosis	28	<5		NC		NC		<5		NC		NC			
Coarctation of aorta	39	<5		NC		NC		<5		NC		NC			
Bicuspid aortic valve	37	<5		NC		NC		<5		NC		NC			
RVOTO	127	<5		NC		NC		<5		NC		NC			
P(v)S	94	<5		NC		NC		<5		NC		NC			
Pulmonary atresia	13	<5		NC		NC		<5		NC		NC			
Septal	459	14	(3.1%)	1.31	0.75-2.30	1.27	0.70-2.28	6	(1.3%)	**3.70**	**1.48-9.25***	**3.06**	**1.14-8.23**		
Perimembranous VSD	91	5	(5.5%)	2.3	0.97-6.08	2.33	0.91-6.00	<5		NC		NC			
Muscular VSD	218	<5		NC		NC		<5		NC		NC			
Other VSD	67	<5		NC		NC		<5		NC		NC			
ASD	82	<5		NC		NC		<5		NC		NC			
AVSD	24	<5		NC		NC		<5		NC		NC			
APVR	17	<5		NC		NC		<5		NC		NC			
Total APVR	11	<5		NC		NC		<5		NC		NC			
Complex	38	<5		NC		NC		<5		NC		NC			
Single ventricle	11	<5		NC		NC		<5		NC		NC			
Associations															
CoA + VSD	14	<5		NC		NC		<5		NC		NC			
P(v)S + VSD	17	<5		NC		NC		<5		NC		NC			

CHD, congenital heart defects; d-TGA, dextro-transposition of the great arteries; LVOTO, left ventricular outflow tract obstruction; HLHS, hypoplastic left heart syndrome; RVOTO, right ventricular outflow tract obstruction; P(v)S, pulmonary (valve) stenosis; CoA, coarctation of aorta; VSD, ventricular septal defect; ASD, atrial septal defect; AVSD, atrioventricular septal defect; APVR, anomalous pulmonary venous return; NC, not calculated due to sparse data. [a] Adjusted for child sex, maternal age at delivery (as continuous variable), education level, maternal BMI (as

continuous variable), smoking and alcohol use during pregnancy, folic acid supplementation, and fertility problems. *p-value <0.01. **Bold indicates significant value (P<0.05)**

Table S3 Prevalence, crude OR and adjusted OR of maternal periconceptional exposure and multiple CHDs in the offspring.

		Occupational exposure													
		Any exposure						Organic dust							
	Total	Exposed		Unadjusted		Adjusted [a]		Exposed		Unadjusted		Adjusted [a]			
CHD classification	n	n	%	OR	95% CI	OR	95% CI	n	%	OR	95% CI	OR	95% CI		
Controls	5602	1992	(35.6%)	Ref		Ref	1992	109	(66.1%)	Ref		Ref			
Total CHD	165	68	(41.1%)	1.27	0.93-1.74	1.23	0.88-1.73	56	(33.9%)	1.27	0.91-1.76	1.37	0.97-1.94		
Conotruncal	30	14	(46.7%)	1.59	0.77-3.26	1.42	0.65-3.10	12	(40.0%)	1.64	0.79-3.42	1.78	0.81-3.89		
d-TGA	10	5	(50.0%)	1.81	0.52-6.27	1.43	0.37-5.54	5	(50.0%)	2.46	0.71-8.52	2.16	0.57-8.22		
Tetralogy of Fallot	15	7	(46.7%)	1.59	0.57-4.38	1.32	0.45-3.92	6	(40.0%)	1.64	0.58-4.62	1.95	0.66-5.72		
LVOTO	18	7	(38.9%)	1.15	0.45-2.98	1.09	0.41-2.89	7	(38.9%)	1.57	0.61-4.05	1.58	0.60-4.14		
RVOTO	12	6	(50.0%)	1.81	0.58-5.63	2.32	0.73-7.40	6	(50.0%)	2.46	0.79-7.65	3.11	0.97-9.99		
Septal	85	30	(35.3%)	0.99	0.63-1.55	0.88	0.54-1.42	21	(24.7%)	0.81	0.49-1.33	0.86	0.52-1.44		
Perimembranous VSD	26	10	(38.5%)	1.13	0.51-2.50	0.93	0.39-2.18	7	(26.9%)	0.91	0.38-2.16	1.04	0.43-2.56		
Muscular VSD	30	9	(30.0%)	0.78	0.36-1.70	0.79	0.35-1.78	6	(20.0%)	0.62	0.25-1.51	0.70	0.28-1.76		
Other VSD	11	5	(45.5%)	1.51	0.46-4.96	1.45	0.40-5.24	<5		NC		NC			
ASD	16	5	(31.3%)	0.82	0.29-2.37	0.78	0.26-2.33	<5		NC		NC			
AVSD	4	<5		NC		NC		<5		NC		NC			
APVR	2	<5		NC		NC		<5		NC		NC			
Complex	7	<5		NC		NC		<5		NC		NC			

CHD, congenital heart defects; d-TGA, dextro-transposition of the great arteries; LVOTO, left ventricular outflow tract obstruction; HLHS, hypoplastic left heart syndrome; RVOTO, right ventricular outflow tract obstruction; P(v)S, pulmonary (valve) stenosis; CoA, coarctation of aorta; VSD, ventricular septal defect; ASD, atrial septal defect; AVSD, atrioventricular septal defect; APVR, anomalous pulmonary venous return; NC, not calculated due to sparse data. [a] Adjusted for child sex, maternal age at delivery (as continuous variable), education level, maternal BMI (as continuous variable), smoking and alcohol use during pregnancy, folic acid supplementation, and fertility problems. *p-value <0.01. Exposure to metal dust and fumes was not calculated due to zero exposed cases. **Bold indicates significant value (P<0.05)**

Table S3 Continued.

		Occupational exposure													
		Mineral dust						Solvents							
	Total	Exposed		Unadjusted		Adjusted [a]		Exposed		Unadjusted		Adjusted [a]			
CHD classification	n	n	%	OR	95% CI	OR	95% CI	n	%	OR	95% CI	OR	95% CI		
Controls	5602	418	(7.5%)	Ref		Ref		1370	(24.5%)	Ref		Ref			
Total CHD	165	16	(9.7%)	1.33	0.79-2.25	1.13	0.64-2.01	37	(22.4%)	0.89	0.62-1.29	0.96	0.66-1.41		
Conotruncal	30	5	(16.7%)	2.48	0.95-6.51	2.01	0.66-6.16	5	(16.7%)	0.62	0.24-1.62	0.70	0.26-1.86		
d-TGA	10	<5		NC		NC		<5		NC		NC			
Tetralogy of Fallot	15	<5		NC		NC		<5		NC		NC			
LVOTO	18	<5		NC		NC		5	(27.8%)	1.19	0.42-3.34	1.19	0.42-3.37		
RVOTO	12	<5		NC		NC		<5		NC		NC			
Septal	85	7	(8.2%)	1.11	0.51-2.43	0.85	0.37-1.94	14	(16.5%)	0.61	0.34-1.08	0.65	0.36-1.18		
Perimembranous VSD	26	<5		NC		NC		5	(19.2%)	0.74	0.28-1.95	0.79	0.29-2.16		
Muscular VSD	30	<5		NC		NC		<5		NC		NC			
Other VSD	11	<5		NC		NC		<5		NC		NC			
ASD	16	<5		NC		NC		<5		NC		NC			
AVSD	4	<5		NC		NC		<5		NC		NC			
APVR	2	<5		NC		NC		<5		NC		NC			
Complex	7	<5		NC		NC		<5		NC		NC			

CHD, congenital heart defects; d-TGA, dextro-transposition of the great arteries; LVOTO, left ventricular outflow tract obstruction; HLHS, hypoplastic left heart syndrome; RVOTO, right ventricular outflow tract obstruction; P(v)S, pulmonary (valve) stenosis; CoA, coarctation of aorta; VSD, ventricular septal defect; ASD, atrial septal defect; AVSD, atrioventricular septal defect; APVR, anomalous pulmonary venous return; NC, not calculated due to sparse data. [a] Adjusted for child sex, maternal age at delivery (as continuous variable), education level, maternal BMI (as continuous variable), smoking and alcohol use during pregnancy, folic acid supplementation, and fertility problems. *p-value <0.01. Exposure to metal dust and fumes was not calculated due to zero exposed cases. **Bold indicates significant value (P<0.05)**

Table S3 Continued.

	Occupational exposure						
	Pesticides						
	Total	Exposed		Unadjusted		Adjusted [a]	
CHD classification	n	n	%	OR	95% CI	OR	95% CI
Controls	5602	131	(2.3%)	Ref		Ref	
Total CHD	165	5	(3.0%)	1.31	0.53-3.23	1.27	0.50-3.24
Conotruncal	30	<5		NC		NC	
d-TGA	10	<5		NC		NC	
Tetralogy of Fallot	15	<5		NC		NC	
LVOTO	18	<5		NC		NC	
RVOTO	12	<5		NC		NC	
Septal	85	<5		NC		NC	
Perimembranous VSD	26	<5		NC		NC	
Muscular VSD	30	<5		NC		NC	
Other VSD	11	<5		NC		NC	
ASD	16	<5		NC		NC	
AVSD	4	<5		NC		NC	
APVR	2	<5		NC		NC	
Complex	7	<5		NC		NC	

CHD, congenital heart defects; d-TGA, dextro-transposition of the great arteries; LVOTO, left ventricular outflow tract obstruction; HLHS, hypoplastic left heart syndrome; RVOTO, right ventricular outflow tract obstruction; P(v)S, pulmonary (valve) stenosis; CoA, coarctation of aorta; VSD, ventricular septal defect; ASD, atrial septal defect; AVSD, atrioventricular septal defect; APVR, anomalous pulmonary venous return; NC, not calculated due to sparse data. [a] Adjusted for child sex, maternal age at delivery (as continuous variable), education level, maternal BMI (as continuous variable), smoking and alcohol use during pregnancy, folic acid supplementation, and fertility problems. *p-value <0.01. Exposure to metal dust and fumes was not calculated due to zero exposed cases. **Bold indicates significant value (P<0.05)**

Table S4 Prevalence, crude OR and adjusted OR of maternal occupational exposure and CHDs in the offspring restricted to non-drinking and non-smoking women.

		Occupational exposure											
		Any exposure						Organic dust					
	Total	Exposed		Unadjusted		Adjusted [a]		Exposed		Unadjusted		Adjusted [a]	
CHD classification	n	n	%	OR	95% CI	OR	95% CI	n	%	OR	95% CI	OR	95% CI
Controls	4622	1625	(35.2%)	Ref		Ref	1992	1341	(29.0%)	Ref		Ref	
Total CHD	703	254	(37.1%)	1.04	0.88-1.23	0.99	0.83-1.18	204	(29.0%)	1.00	0.84-1.19	1.00	0.83-1.20
Conotruncal	92	36	(39.1%)	1.19	0.78-1.81	1.17	0.75-1.82	31	(33.7%)	1.24	0.80-1.93	1.24	0.79-1.95
d-TGA	74	16	(40.0%)	1.23	0.65-2.32	1.23	0.63-2.42	13	(32.5%)	1.18	0.61-2.29	1.18	0.59-2.36
Tetralogy of Fallot	32	13	(40.6%)	1.26	0.62-2.56	1.34	0.64-2.80	12	(37.5%)	1.47	0.72-3.01	1.58	0.76-3.29
LVOTO	105	32	(30.5%)	0.81	0.53-1.23	0.71	0.45-1.10	27	(25.7%)	0.85	0.54-1.32	0.82	0.52-1.30
HLHS	32	13	(40.6%)	1.26	0.62-2.56	0.99	0.47-2.08	11	(34.4%)	1.28	0.62-2.67	1.05	0.49-2.26
Aortic stenosis	21	<5		NC		NC		<5		NC		NC	
Coarctation of aorta	19	6	(31.6%)	0.85	0.32-2.24	0.67	0.23-1.92	5	(26.3%)	0.87	0.31-2.43	0.95	0.33-2.71
Bicuspid aortic valve	26	7	(26.9%)	0.68	0.29-1.62	0.59	0.24-1.45	6	(23.1%)	0.73	0.29-1.83	0.71	0.28-1.79
RVOTO	88	40	(45.5%)	**1.54**	**1.01-2.35**	1.43	0.92-2.21	34	(38.6%)	1.54	0.99-2.38	1.48	0.95-2.31
P(v)S	64	32	(50.0%)	**1.84**	**1.13-3.02**	1.70	1.02-2.83	27	(42.2%)	**1.79**	**1.08-2.94**	**1.70**	**1.02-2.83**
Pulmonary atresia	8	<5		NC		NC		<5		NC		NC	
Septal	333	114	(34.2%)	0.96	0.76-1.21	0.90	0.70-1.15	86	(25.8%)	0.85	0.66-1.10	0.86	0.66-1.12
Perimembranous VSD	74	28	(37.8%)	1.12	0.70-1.80	1.05	0.64-1.73	21	(28.4%)	0.97	0.58-1.61	1.01	0.60-1.70
Muscular VSD	157	50	(31.8%)	0.86	0.61-1.21	0.86	0.61-1.23	40	(25.5%)	0.84	0.58-1.20	1.88	0.60-1.28
Other VSD	40	13	(32.5%)	0.89	0.46-1.73	0.86	0.43-1.73	9	(22.5%)	0.71	0.34-1.50	0.72	0.34-1.55
ASD	60	23	(38.3%)	1.15	0.70-1.94	0.97	0.57-1.66	16	(26.7%)	0.89	0.50-1.58	0.82	0.45-1.46
AVSD	28	7	(25.0%)	0.60	0.26-1.42	0.67	0.27-1.63	6	(21.4%)	0.67	0.27-1.66	0.81	0.32-2.06
APVR	9	<5		NC		NC		<5		NC		NC	
Complex	24	9	(37.5%)	1.11	0.48-2.53	1.16	0.48-2.79	7	(29.2%)	1.01	0.42-2.44	1.10	0.44-2.75
Single ventricle	8	<5		NC		NC		<5		NC		NC	
Associations													
CoA + VSD	7	<5		NC		NC		<5		NC		NC	
P(v)S + VSD	10	6	(60.0%)	2.77	0.77-9.82	3.43	0.95-12.43	5	(50.0%)	2.45	**0.90-5.47**	**2.83**	**0.81-9.90**

CHD, congenital heart defects; d-TGA, dextro-transposition of the great arteries; LVOTO, left ventricular outflow tract obstruction; HLHS, hypoplastic left heart syndrome; RVOTO, right ventricular outflow tract obstruction; P(v)S, pulmonary (valve) stenosis; CoA, coarctation of aorta; VSD, ventricular septal defect; ASD, atrial septal defect; AVSD, atrioventricular septal defect; APVR, anomalous pulmonary venous return; NC, not calculated due to sparse data. [a] Adjusted for child sex, maternal age at delivery (as continuous variable), education level, maternal BMI (as continuous variable), folic acid supplementation, and fertility problems. *p-value <0.01. **Bold indicates significant value (P<0.05)**

Table S4 Continued.

		Occupational exposure													
		Mineral dust						Solvents							
	Total	Exposed		Unadjusted		Adjusted [a]		Exposed		Unadjusted		Adjusted [a]			
CHD classification	n	n	%	OR	95% CI	OR	95% CI	n	%	OR	95% CI	OR	95% CI		
Controls	4622	312	(6.8%)	Ref		Ref		1151	(24.0%)	Ref		Ref			
Total CHD	703	63	(9.0%)	**1.36**	**1.02-1.81**	1.87	0.94-1.73	169	(24.9%)	0.95	0.79-1.15	0.93	0.77-1.13		
Conotruncal	92	10	(10.9%)	1.69	0.87-3.28	1.67	0.83-3.36	23	(25.0%)	1.01	0.62-1.62	1.04	0.64-1.69		
d-TGA	74	5	(12.5%)	1.97	0.77-5.07	2.03	0.75-5.47	9	(22.5%)	0.88	0.42-1.84	0.99	0.46-2.12		
Tetralogy of Fallot	32	<5		NC		NC		9	(28.1%)	1.18	0.54-2.56	1.19	0.54-2.63		
LVOTO	105	8	(7.6%)	1.14	0.55-2.36	1.08	0.51-2.32	21	(20.0%)	0.75	0.47-1.22	0.70	0.42-1.16		
HLHS	32	<5		NC		NC		9	(28.1%)	1.18	0.54-2.56	0.90	0.40-2.05		
Aortic stenosis	21	<5		NC		NC		<5		NC		NC			
Coarctation of aorta	19	<5		NC		NC		<5		NC		NC			
Bicuspid aortic valve	26	<5		NC		NC		6	(23.1%)	0.91	0.36-2.26	0.88	0.35-2.25		
RVOTO	88	14	(15.9%)	**1.61**	**1.46-4.68***	**2.36**	**1.26-4.40**	26	(29.5%)	1.27	0.80-2.01	1.19	0.74-1.91		
P(v)S	64	11	(17.2%)	**2.87**	**1.48-5.54***	**2.46**	**1.21-4.97**	20	(31.3%)	1.37	0.81-2.34	1.32	0.77-2.27		
Pulmonary atresia	8	<5		NC		NC		<5		NC		NC			
Septal	333	27	(8.1%)	1.22	0.81-1.84	1.10	0.71-1.70	74	(22.2%)	0.86	0.66-1.13	0.85	0.64-1.12		
Perimembranous VSD	74	5	(6.8%)	1.00	0.40-2.50	0.90	0.35-2.33	20	(27.0%)	1.12	0.67-1.87	1.14	0.67-1.94		
Muscular VSD	157	16	(10.2%)	1.57	0.92-2.66	1.55	0.88-2.74	30	(19.1%)	0.71	0.48-1.07	0.73	0.48-1.09		
Other VSD	40	<5		NC		NC		8	(20.0%)	0.75	0.35-1.64	0.75	0.34-1.65		
ASD	60	<5		NC		NC		16	(26.7%)	1.10	0.62-1.95	0.98	0.54-1.75		
AVSD	28	<5		NC		NC		<5		NC		NC			
APVR	9	<5		NC		NC		<5		NC		NC			
Complex	24	<5		NC		NC		7	(29.2%)	1.24	0.51-3.00	1.27	0.51-3.17		
Single ventricle	8	<5		NC		NC		<5		NC		NC			
Associations															
CoA + VSD	7	<5		NC		NC		<5		NC		NC			
P(v)S + VSD	10	<5		NC		NC		5	(50.0%)	3.02	0.87-10.44	3.30	0.94-11.58		

CHD, congenital heart defects; d-TGA, dextro-transposition of the great arteries; LVOTO, left ventricular outflow tract obstruction; HLHS, hypoplastic left heart syndrome; RVOTO, right ventricular outflow tract obstruction; P(v)S, pulmonary (valve) stenosis; CoA, coarctation of aorta; VSD, ventricular septal defect; ASD, atrial septal defect; AVSD, atrioventricular septal defect; APVR, anomalous pulmonary venous return; NC, not calculated due to sparse data. [a] Adjusted for child sex, maternal age at delivery (as continuous variable), education level, maternal BMI (as continuous variable), folic acid supplementation, and fertility problems. *p-value <0.01. **Bold indicates significant value (P<0.05)**

Table S4 Continued.

		Occupational exposure												
		Pesticides						Metal dust and fumes						
	Total	Exposed		Unadjusted		Adjusted [a]		Exposed		Unadjusted		Adjusted [a]		
CHD classification	n	n	%	OR	95% CI	OR	95% CI	n	%	OR	95% CI	OR	95% CI	
Controls	4622	104	(2.3%)	Ref		Ref		11	(0.2%)	Ref		Ref		
Total CHD	703	23	(3.3%)	1.47	0.93-2.33	1.35	0.83-2.18	8	(1.1%)	**4.83**	**1.93-12.04**	**3.84**	**1.38-10.65**	
Conotruncal	92	<5		NC		NC		<5		NC		NC		
d-TGA	74	<5		NC		NC		<5		NC		NC		
Tetralogy of Fallot	32	<5		NC		NC		<5		NC		NC		
LVOTO	105	<5		NC		NC		<5		NC		NC		
HLHS	32	<5		NC		NC		<5		NC		NC		
Aortic stenosis	21	<5		NC		NC		<5		NC		NC		
Coarctation of aorta	19	<5		NC		NC		<5		NC		NC		
Bicuspid aortic valve	26	<5		NC		NC		<5		NC		NC		
RVOTO	88	<5		NC		NC		<5		NC		NC		
P(v)S	64	<5		NC		NC		<5		NC		NC		
Pulmonary atresia	8	<5		NC		NC		<5		NC		NC		
Septal	333	14	(4.2%)	**1.91**	**1.08-3.37**	**1.71**	**0.94-3.11**	<5		NC		NC		
Perimembranous VSD	74	5	(6.8%)	**3.15**	**1.24-7.97**	**2.92**	**1.11-7.67**	<5		NC		NC		
Muscular VSD	157	5	(3.2%)	1.43	0.57-3.56	1.37	0.53-3.51	<5		NC		NC		
Other VSD	40	<5		NC		NC		<5		NC		NC		
ASD	60	<5		NC		NC		<5		NC		NC		
AVSD	28	<5		NC		NC		<5		NC		NC		
APVR	9	<5		NC		NC		<5		NC		NC		
Complex	24	<5		NC		NC		<5		NC		NC		
Single ventricle	8	<5		NC		NC		<5		NC		NC		
Associations														
CoA + VSD	7	<5		NC		NC		<5		NC		NC		
P(v)S + VSD	10	<5		NC		NC		<5		NC		NC		

CHD, congenital heart defects; d-TGA, dextro-transposition of the great arteries; LVOTO, left ventricular outflow tract obstruction; HLHS, hypoplastic left heart syndrome; RVOTO, right ventricular outflow tract obstruction; P(v)S, pulmonary (valve) stenosis; CoA, coarctation of aorta; VSD, ventricular septal defect; ASD, atrial septal defect; AVSD, atrioventricular septal defect; APVR, anomalous pulmonary venous return; NC, not calculated due to sparse data. [a] Adjusted for child sex, maternal age at delivery (as continuous variable), education level, maternal BMI (as continuous variable), folic acid supplementation, and fertility problems. *p-value <0.01. **Bold indicates significant value (P<0.05)**

Table S5 Prevalence, crude OR and adjusted OR of maternal occupational exposure and CHDs in the offspring.

	Controls (n=5602)		CHDs (n=1174)		Unadjusted		Adjusted [a]	
	n	(%)	n	(%)	OR	95% CI	OR	95% CI
Any exposure								
No exposure	3611	(64.5%)	733	(62.4%)	Ref		Ref	
Low exposure	1802	(32.2%)	386	(32.9%)	1.06	0.92-1.21	1.00	0.86-1.16
High exposure	189	(3.4%)	55	(4.7%)	**1.43**	**1.05-1.96**	1.37	0.97-1.94

CHD, congenital heart defects. [a] Adjusted for child sex, maternal age at delivery (as continuous variable), educational level, maternal BMI (as continuous variable), smoking and alcohol use during pregnancy, folic acid supplementation, and fertility problems. **Bold indicates significant value (P<0.05)**
